# Rule-Based Models of the Interplay between Genetic and Environmental Factors in Childhood Allergy

**DOI:** 10.1371/journal.pone.0080080

**Published:** 2013-11-19

**Authors:** Susanne Bornelöv, Annika Sääf, Erik Melén, Anna Bergström, Behrooz Torabi Moghadam, Ville Pulkkinen, Nathalie Acevedo, Christina Orsmark Pietras, Markus Ege, Charlotte Braun-Fahrländer, Josef Riedler, Gert Doekes, Michael Kabesch, Marianne van Hage, Juha Kere, Annika Scheynius, Cilla Söderhäll, Göran Pershagen, Jan Komorowski

**Affiliations:** 1 Science for Life Laboratory, Department of Cell and Molecular Biology, Uppsala University, Uppsala, Sweden; 2 Institute of Environmental Medicine, Karolinska Institutet, Stockholm, Sweden; 3 Sachs' Children's Hospital, South General Hospital, Stockholm, Sweden; 4 Research Programs Unit, Biomedicum, University of Helsinki and Folkhälsan Institute of Genetics, Helsinki, Finland; 5 Department of Biosciences and Nutrition, Karolinska Institutet, Stockholm, Sweden; 6 Department of Medicine Solna, Translational Immunology Unit, Karolinska Institutet and University Hospital, Stockholm, Sweden; 7 Dr von Hauner Children's Hospital, Ludwig-Maximilians-University, Munich, Germany; 8 Department of Epidemiology and Public Health, Swiss Tropical and Public Health Institute, Basel, Switzerland; 9 Department of Public Health, University of Basel, Basel, Switzerland; 10 Department of Children and Young Adults' Medicine, Kardinal Schwarzenberg Hospital, Schwarzach, Austria; 11 Institute for Risk Assessment Sciences, Utrecht University, Utrecht, The Netherlands; 12 Department of Pediatric Pneumology, Allergy and Neonatology, Hannover Medical School, Hannover, Germany; 13 Clinical Immunology and Allergy Unit, Department of Medicine Solna, Karolinska Institutet and University Hospital, Stockholm, Sweden; 14 Science for Life Laboratory, Karolinska Institutet, Stockholm, Sweden; 15 Interdisciplinary Centre for Mathematical and Computational Modelling, University of Warsaw, Warsaw, Poland; Memorial Sloan Kettering Cancer Center, United States of America

## Abstract

Both genetic and environmental factors are important for the development of allergic diseases. However, a detailed understanding of how such factors act together is lacking. To elucidate the interplay between genetic and environmental factors in allergic diseases, we used a novel bioinformatics approach that combines feature selection and machine learning. In two materials, PARSIFAL (a European cross-sectional study of 3113 children) and BAMSE (a Swedish birth-cohort including 2033 children), genetic variants as well as environmental and lifestyle factors were evaluated for their contribution to allergic phenotypes. Monte Carlo feature selection and rule based models were used to identify and rank rules describing how combinations of genetic and environmental factors affect the risk of allergic diseases. Novel interactions between genes were suggested and replicated, such as between *ORMDL3* and *RORA*, where certain genotype combinations gave odds ratios for current asthma of 2.1 (95% CI 1.2-3.6) and 3.2 (95% CI 2.0-5.0) in the BAMSE and PARSIFAL children, respectively. Several combinations of environmental factors appeared to be important for the development of allergic disease in children. For example, use of baby formula and antibiotics early in life was associated with an odds ratio of 7.4 (95% CI 4.5-12.0) of developing asthma. Furthermore, genetic variants together with environmental factors seemed to play a role for allergic diseases, such as the use of antibiotics early in life and *COL29A1* variants for asthma, and farm living and *NPSR1* variants for allergic eczema. Overall, combinations of environmental and life style factors appeared more frequently in the models than combinations solely involving genes. In conclusion, a new bioinformatics approach is described for analyzing complex data, including extensive genetic and environmental information. Interactions identified with this approach could provide useful hints for further in-depth studies of etiological mechanisms and may also strengthen the basis for risk assessment and prevention.

## Introduction

Allergic diseases, including asthma, rhinitis and eczema, are complex chronic disorders showing an increased prevalence over recent decades [1,2]. Twin and family studies have demonstrated the importance of the genetic architecture in allergic disease [3] and candidate-gene association studies have revealed a large number of asthma, eczema and atopy susceptibility genes [4,5]. Furthermore, genome-wide association (GWA) studies have identified new loci associated with epidermal damage, immune dysregulation and inflammation in the pathogenesis of asthma [6,7] and eczema [8,9]. However, genetic associations alone cannot explain the time trends in development of allergy, which must relate to changes in lifestyle and environmental exposures. For example, maternal smoking and farming exposures during pregnancy affect the risk for childhood asthma, suggesting that exposures already *in utero* are of importance [10,11]. Also, living on a farm during the first years of life has been associated with protection from allergic diseases [12-15]. Other risk factors for asthma include obesity and air pollution exposure [16,17]. Moreover, the prevalence of atopy is lower in children with an anthroposophic upbringing corresponding to a lifestyle that is characterized by consumption of biodynamic food and restricted use of antibiotics, antipyretics and vaccinations as well as several other life style features [18,19].

It is evident that complex diseases, such as asthma and allergy, develop as a result of interactions between genes and the environment. Toll-like receptor 2 (TLR2), for instance, has been shown to affect the risk of asthma and atopy in farmers [20], and *CD14* appears to modify the effect of farm milk on allergic disease [21]. Gene-environment interaction studies are also emerging on a genomic scale, including studies on childhood asthma and farming exposures [22]. Importantly, there are still many challenges when taking interaction studies to the genome-level and there is a need for new analysis tools for interpretation of complex datasets. 

Machine learning methods have become increasingly popular in the study of complex interactions, including those in asthma and allergy. Previous applications include clustering of children by response to common allergens [23], or of allergens with respect to antibody response [24], prediction of allergenicity in proteins [25], or of severe asthma exacerbations using single nucleotide polymorphisms (SNPs) from GWAS [26], as well as, examination of asthma susceptibility regions [27]. In this study, we have used a new approach by combining feature selection and classification to model asthma and allergy phenotypes based on genetic and environmental factors. The primary aim was to apply this new methodology in exploratory analyses to assess the interplay between existing data on genotype, lifestyle and environmental exposure in two well-characterized European datasets, the BAMSE and PARSIFAL studies. To our knowledge, this methodology has not been applied before to assess gene-gene or gene-environment interactions for allergy in children. We believe this approach will be of great use also in many other research fields that are lacking advanced tools for analyzing large complex datasets.

## Materials and Methods

### Ethics Statement

The BAMSE study was approved by the Ethics Committee of Karolinska Institutet, Stockholm, Sweden. The PARSIFAL study included children from five European countries and was approved by Ethics Committees in each country. The ethical approvals specifically referred to genetic analyses. Written informed consent was obtained from the parents and/or legal guardians. All biosamples were assigned a code and treated anonymously.

### Study Populations

**BAMSE** is a prospective Swedish birth cohort, where newborn infants were recruited 1994-1996 and questionnaire data about baseline study characteristics were obtained from 4,089 children [28,29]. Parents answered questionnaires on the children’s symptoms related to allergy and lifestyle factors at approximately age 1, 2, 4 and 8 years. At the 4- and 8-year follow-up, blood samples were drawn from 2,614 and 2,480 children, respectively. This study includes DNA extracted from 2,033 blood samples (1,051 boys and 982 girls) (Table 1).

**Table 1:** Overview of the epidemiologic studies BAMSE and PARSIFAL.

	**BAMSE**		**PARSIFAL**	
Total number	2033		3113	
Boys (%)	52		51	
Age (years; average)	8.3		9.0	
Phenotypes (n = count)	Affected	Unaffected	Affected	Unaffected
Asthma	293	1661	261	2801
Allergic asthma	158	1123	144	2058
Non-allergic asthma	135	1123	117	2058
Current asthma	131	1568	119	2663
Wheeze	226	1796	236	2849
Eczema	182	1775	399	2650
Allergic eczema	98	1190	190	1960
Non-allergic eczema	84	1190	209	1960
Rhinoconjunctivitis	313	1714	215	2868
Atopic sensitization >3.5 kU/l	349	1682	487	2625
Atopic sensitization >0.35 kU/l	717	1314	896	2214

**PARSIFAL** is a cross-sectional study including 5-13 year old children from 5 different European countries [12]. The study was originally designed to investigate lifestyle and environmental factors in farm children, Steiner school children, and corresponding reference groups. This study includes 3113 children with available DNA from blood (1,579 boys and 1,534 girls) (Table 1).

### Definition of Exposures

This study primarily used information on different exposures related to farming and an anthroposophic life style from parental questionnaires regarding children in the PARSIFAL study. The overall response rate to the questionnaire was 69% with country specific rates ranging from 50% in the Netherlands to 82% in Switzerland [12]. Questions on exposures and lifestyle factors related to living on a farm were based on an earlier study in Switzerland, Germany and Austria [41] while questions regarding factors associated with the anthroposophic lifestyle originated from a Swedish study [18]. In BAMSE exposure and life style information was provided in a parental questionnaire when the children were about three months [29]. Around 75% of all children born in predefined areas of Stockholm county were included.

### Definition of Phenotypes

***Asthma*** was defined as doctor’s diagnosis of asthma ever up to 8 years in BAMSE and up to 13 years (median 9 years) in PARSIFAL. ***Currentasthma*** was defined as *asthma* in combination with at least one episode of wheezing during the last 12 months prior to the questionnaire date. ***Allergicasthma*** was defined as having *asthma*, in combination with *atopic sensitization*, i.e. allergen-specific serum IgE ≥ 0.35 kU/liter against inhalant and/or food allergens, while ***non-allergicasthma*** was defined as having *asthma* without raised allergen-specific serum IgE levels. The same reference group was used for allergic and non-allergic asthma, including only children that did not have asthma and were not sensitized. ***Wheeze*** was defined as at least one episode of wheezing during the last 12 months prior to the questionnaire date. ***Eczema*** was defined as doctor’s diagnosis of eczema at age 4-9 years in BAMSE, and as doctor’s diagnosis of atopic eczema ever prior to the date of the questionnaire in PARSIFAL. ***Allergiceczema*** was defined as having *eczema*, in combination with *atopic sensitization*, i.e. allergen-specific serum IgE ≥ 0.35 kU/liter against inhalant and/or food allergens, while ***non-allergiceczema*** was defined as having *eczema* without raised allergen-specific serum IgE levels. The reference group was composed of non-eczema and non-sensitized children. ***Rhinoconjunctivitis*** was defined as prolonged sneezing or runny nose or nasal block-up during the last 12 months prior to the date of questionnaire. ***Atopicsensitization*** was defined as having allergen-specific serum IgE (≥ 0.35 kU/L) against a mixture of common airborne allergens (Phadiatop^®^) and/or common food allergens (fx5^®^) (ImmunoCAP^TM^, Phadia AB, Uppsala, Sweden). A more strict definition for *Atopic sensitization* was also used (IgE ≥ 3.5 kU/L). 

### Genotypes and Environmental Factors

BAMSE and PARSIFAL have been used in several previous genetic studies, and SNPs in 29 susceptibility genes for childhood allergies have been genotyped in these datasets (Table S2 and Methods S1 for detailed genotyping description). In this study, all available genotype data in PARSIFAL (except GWA data) and corresponding data in BAMSE were included for assessment of gene-gene and gene-environment interactions. The environmental factors included are described in Table S1.

### Data Analysis

**Figure 1:**
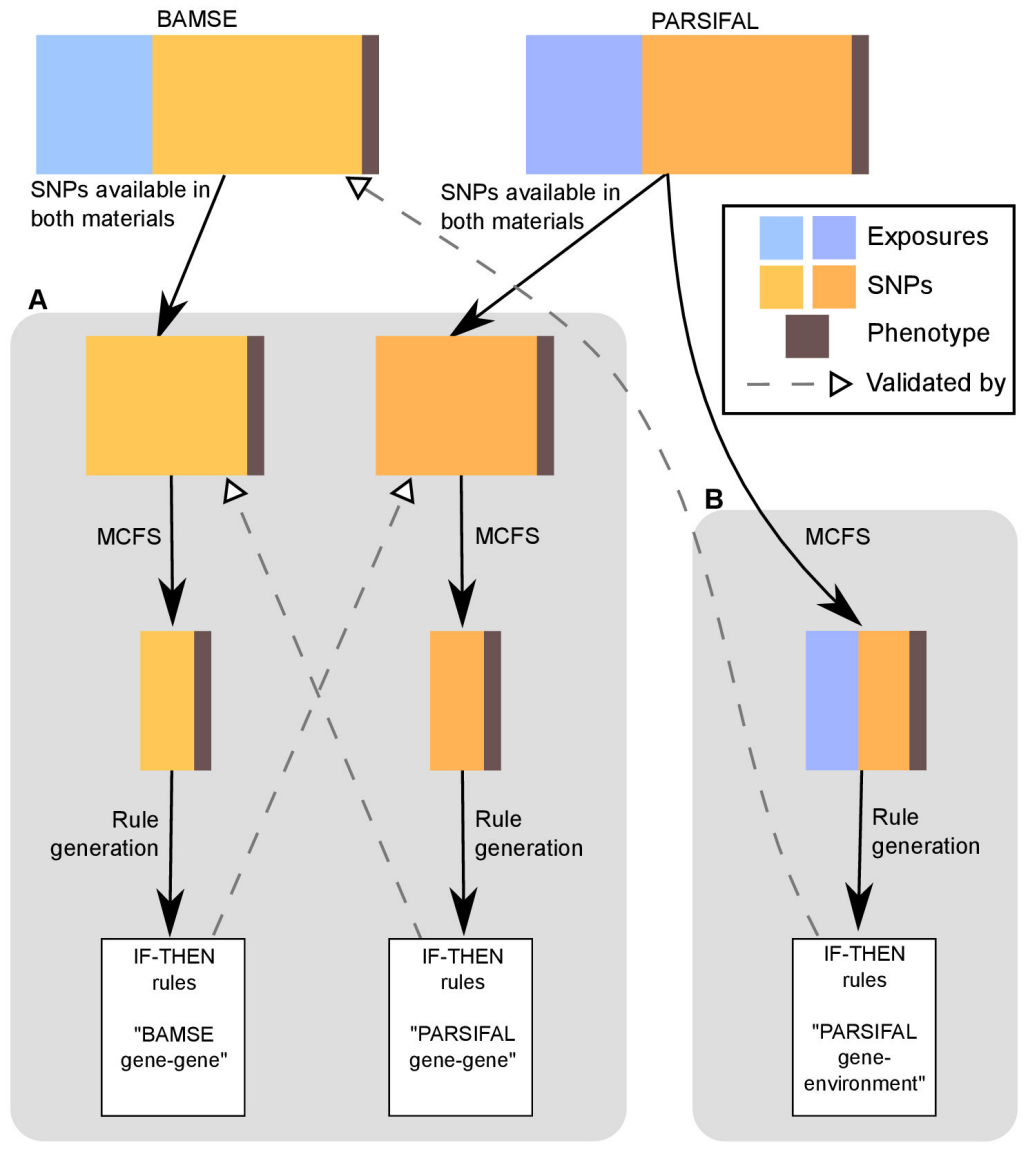
Analysis methodology for factors related to childhood allergy in the epidemiologic studies BAMSE and PARSIFAL. Allergy phenotypes were modeled based on genetic and exposure data to identify (**A**) rules using gene and (**B**) gene and environment data. MCFS selected significant predictors of a phenotype, which was used to generate rules by ROSETTA. First model used 110 SNPs in BAMSE and PARSIFAL, while the second model included both genetic and exposure data in PARSIFAL, using BAMSE for validation when applicable.

The following section is a short summary of the methodology (a detailed description is given in the Methods S1). Feature selection and classification were combined to model the phenotypes based on genetic and/or environmental factors. Each of the 11 phenotypes was analyzed separately. Two different types of models were constructed; the first model aimed at finding *gene-gene* interactions and was based on only those SNPs that were genotyped in both PARSIFAL and BAMSE, and the second model aimed at finding *gene-environment* interactions based on genetic, lifestyle and environmental exposure data available in the two materials. Data on key environmental exposures such as farming life style and detailed use of antibiotics was not available in BAMSE and analyses of *gene-environment* interactions were restricted to PARSIFAL for such exposures. Monte Carlo Feature Selection (MCFS) was used to identify significant predictors of a phenotype [30]. This was followed by model construction using the ROSETTA rough set software [31,32], which describes combinations of factors related to a specific phenotype. The models or “rules” generated by ROSETTA (http://www.lcb.uu.se/tools/rosetta) are easy to read in the form of “IF-THEN” rules. For example, “**IF** mother had asthma **AND** child used antibiotics during first year of life **THEN** the child is predicted to have asthma”. An overview of the methodology is shown in Figure 1 and described in detail in the Methods S1.

Logistic regression was used to estimate associations identified by ROSETTA between genetic/environmental factors and allergic outcomes. The results are presented as odds ratios (ORs) and 95% confidence intervals (CI) using STATA 11 software package (College Station, TX, USA). 

## Results

### Interplay Between Genes in Allergic Diseases

**Figure 2:**
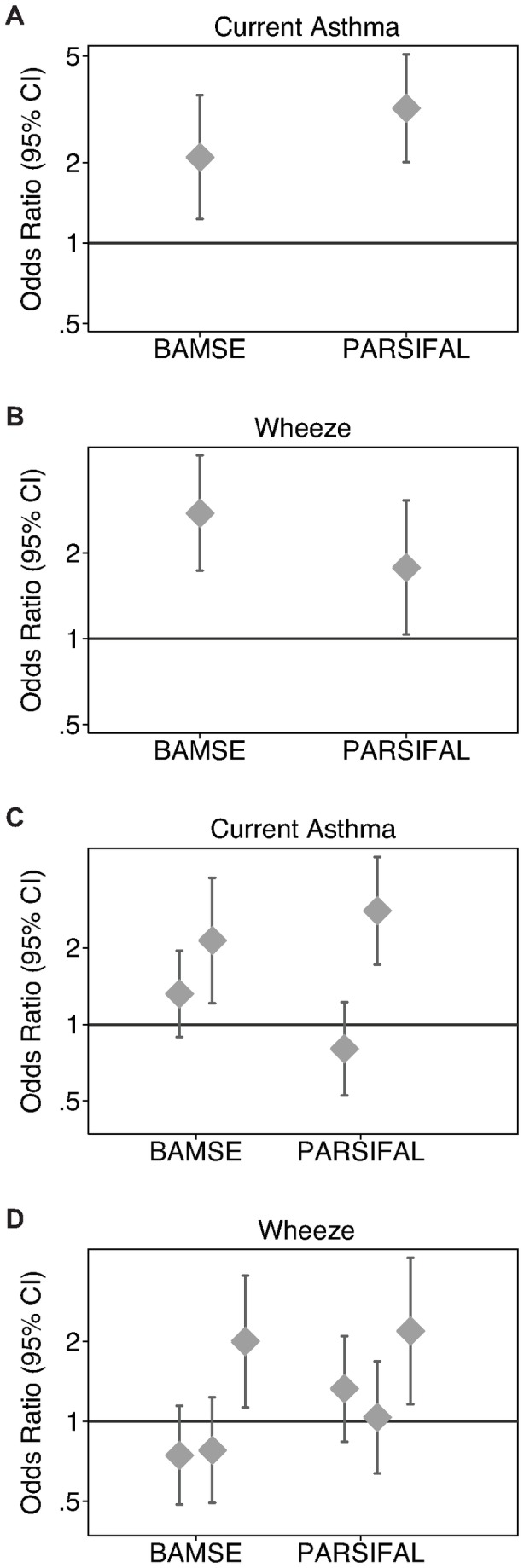
SNP combinations with relevance for current asthma and wheeze in BAMSE and PARSIFAL. The combination of specific genetic variants in (**A**) *ORMDL3-RORA* increases the risk for current asthma^1^, and in (**B**) *ORMDL3-RORA-COL29A1* increase the risk for wheeze^2^. The risk for current asthma and wheeze increased with the number of risk genotypes described by corresponding rule (**C**-**D**). ORs and 95% confidence interval are shown. The major allele count is indicated for each gene below i.e. describing 0, 1 or 2 copies of the major allele. The reference category includes children who do not fulfill the rule. ^1^ IF ORMDL3_rs2305480=2[GG] AND RORA_rs17270362=1[AG] THEN current asthma. ^2^ IF COL29A1_rs11917356=2[AA] AND ORMDL3_rs7216389=0[TT] AND RORA_rs17270362=1[AG] THEN wheeze.

Feature selection and classification were performed in BAMSE and PARSIFAL based on 110 SNPs representing asthma and allergy susceptibility genes. One model was generated for each phenotype, in each material, which resulted in 22 different datasets (Table 2). MCFS was utilized to identify significant SNPs, and on average, 11.1 SNPs were used for the rule generation in ROSETTA. The number of rules, for each phenotype, varied between 3 and 184 (on average 51 rules) and the average accuracy was 55.4%. Of the 39 rules that were significant (p<0.05; hypergeometric distribution; Bonferroni-corrected p-value) 31 (79 %) showed an effect in the same direction in the other material (Table 2; Table S3). Interestingly, novel interactions between SNPs, within a gene (e.g. *RORA*) or between genes (e.g. *RORA* and *ORMDL3*), were indicated by the top-scored rules. The combination of specific genetic variants in *ORMDL3* and *RORA* increased the risk for *current asthma*, with ORs of 2.1 (CI 1.2-3.6) and 3.2 (CI 2.0-5.0) among the BAMSE and PARSIFAL children, respectively (Figure 2 A). Furthermore, a combination of genetic variants in *ORMDL3*, *RORA* and *COL29A1* was associated with *wheeze* in both BAMSE (OR=2.8 (CI 1.7-4.4)) and PARSIFAL (OR= 1.8 (CI 1.0-3.1)) (Figure 2 B). Notably, dose-response analysis could further show that the risk of developing *current asthma* or *wheeze* increased with the number of risk alleles described by these rules (Figure 2 C-D).

**Table 2:** Summary of the analyses on combinations of genetic variants using MCFS and rule generation in BAMSE (n=2033) and PARSIFAL (n=3113).

**Outcome**	**Material**	**Factors**	**Cover.**	**Accur.**	**Rules**	**Val. rules**	**Valid.**
Allergic asthma	BAMSE	13	92.3%	57.0%	61	4	3
	PARSIFAL	8	79.6%	53.4%	18	0	0
Non-allergic asthma	BAMSE	16	92.7%	58.2%	70	2	2
	PARSIFAL	10	84.9%	56.3%	37	1	1
Asthma	BAMSE	9	47.2%	56.6%	21	2	2
	PARSIFAL	17	95.3%	52.0%	111	1	1
Current asthma	BAMSE	12	76.4%	56.0%	34	3	3
	PARSIFAL	9	94.4%	56.5%	53	4	3
Atopic sensitization >3.5 kU/L	BAMSE	4	4.2%	67.7%	3	1	1
	PARSIFAL	6	18.7%	47.5%	3	1	1
Atopic sensitization >0.35 kU/L	BAMSE	18	93.6%	50.2%	124	1	0
	PARSIFAL	21	93.9%	49.2%	184	0	0
Allergic eczema	BAMSE	5	33.2%	57.1%	11	1	1
	PARSIFAL	8	46.2%	56.9%	29	1	1
Eczema	BAMSE	8	49.8%	54.5%	17	0	0
	PARSIFAL	11	73.2%	56.2%	45	3	2
Non-allergic eczema	BAMSE	10	92.0%	56.1%	41	2	0
	PARSIFAL	7	23.5%	58.8%	7	2	2
Rhinoconjunctivitis	BAMSE	9	42.4%	47.5%	18	0	0
	PARSIFAL	5	16.4%	61.5%	7	1	1
Wheeze	BAMSE	18	90.5%	57.4%	121	6	5
	PARSIFAL	21	93.8%	52.9%	106	3	2
**Average**		**11.1**	**65.2%**	**55.4%**	**51.0**	**1.8**	**1.4**

Eleven allergy phenotypes were modeled by combining Monte Carlo feature selection (MCFS) and rule generation using 110 SNPs in BAMSE and PARSIFAL. An overview of the number of significant factors (Factors) identified by MCFS and the estimated model coverage (Cover) and accuracy (Accur), i.e., the quality of the rules, is shown (described in the Methods S1). “Rules”=Total number of rules, “Val.Rules”=rules used for validation and “Valid”=rules that passed validation.

### Interplay Between Genes, Environment and Life Style in Allergic Diseases

Genetic, lifestyle and environmental factors were used to generate models for 11 phenotypes in PARSIFAL, and, when applicable, rules were validated in BAMSE. Data for 188 SNPs and 33 lifestyle and environmental factors were analyzed (Table S1+S2). An average of 15 factors was identified by MCFS as significant predictors of a phenotype (Table 3). Based on these top-ranked factors, ROSETTA generated between 3 and 83 rules, describing “affected” or “unaffected” children with respect to the studied phenotype. From the total of 560 rules, identified for all the 11 phenotypes, 143 rules contained factors that could be validated in the other data set (i.e. BAMSE). The cross-material validation was overall successful, and in total 132 of the 143 PARSIFAL rules (92.3%) showed an effect in the same direction in BAMSE. The rule-based classification had the best performance for allergic eczema, but results are shown here for asthma and atopic sensitization, as well. 

**Table 3:** Summary of the analyses on combinations of genetic variants and environmental factors using MCFS and rule generation in PARSIFAL (n=3113).

**Outcome**	**Factors**	**Cover.**	**Accur.**	**Rules**	**Val. rules**	**Valid.**
Allergic asthma	20	94.1%	61.2%	73	20	18
Asthma	16	93.2%	62.6%	66	16	14
Non-allergic asthma	16	95.8%	64.0%	72	16	16
Current asthma	19	93.0%	63.2%	39	12	10
Atopic sensitization >3.5 kU/L	14	88.4%	64.0%	51	7	7
Atopic sensitization >0.35 kU/L	3	17.3%	59.0%	3	0	0
Allergic eczema	24	95.0%	67.4%	83	17	17
Eczema	11	65.3%	61.7%	30	17	15
Non-allergic eczema	8	69.5%	59.7%	43	9	9
Rhinoconjunctivitis	18	87.1%	63.8%	41	14	13
Wheeze	16	82.1%	60.8%	59	15	13
**Average**	**15**	**80.1%**	**62.5%**	**50.9**	**13**	**12**

Eleven allergy phenotypes were modeled by combining Monte Carlo feature selection (MCFS) and rule generation using genetic and environmental/lifestyle factors in PARSIFAL. An overview of the number of significant factors (Factors) identified by MCFS and the estimated model coverage (Cover) and accuracy (Accur), i.e., the quality of the rules, is shown (described in the Methods S1). “Rules”=Total number of rules, “Val.Rules”=rules used for validation and “Valid”=rules that passed validation.

**Figure 3:**
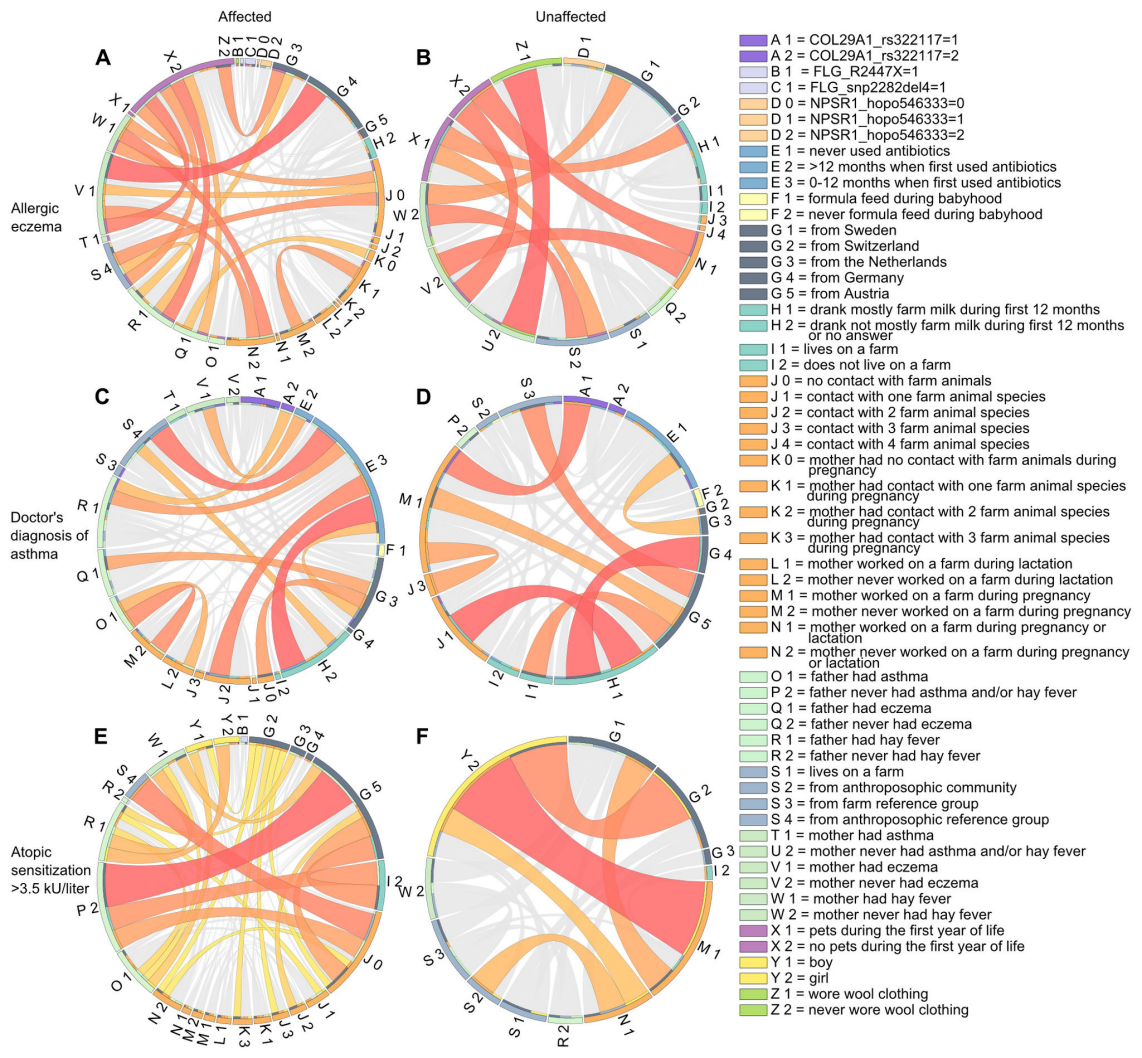
Visualization of co-occurring factors in rules for allergic eczema, asthma and atopic sensitization in PARSIFAL. Rule networks for (**A**-**B**) allergic eczema, (**C**-**D**) asthma and (**E**-**F**) atopic sensitization; affected and unaffected, respectively. Conditions that occur in the rules are on the outer ring, and co-occurrences of conditions in the rules are illustrated by ribbons across the circle connecting the conditions. The ribbon color indicates high (red) to low (grey) scores. The width of the edges is proportional to the number of correctly classified children.

Rule networks were used to visualize genetic and life style factors (rule conditions) that often co-occurred in the rules (Figure 3 A-F). The rule conditions are placed on the circle, and two conditions are connected if they co-occur in at least one rule. The ribbon connecting them is formatted by color and width, depending on the rule quality score and the number of co-occurrences (see Methods S1). For example, in Figure 3 A, “mother’s eczema” (node V1) and “child had no contact with farm animals” (node J0) are connected, visualizing two co-predictors of allergic eczema. The rule networks can be used as a complement to the top-scoring rules to identify frequent combinations (possible two-way interactions). However, not all connections in the figures are the result of an interaction effect, and the existence of an interaction has to be explicitly tested.

#### Allergic eczema

**Figure 4:**
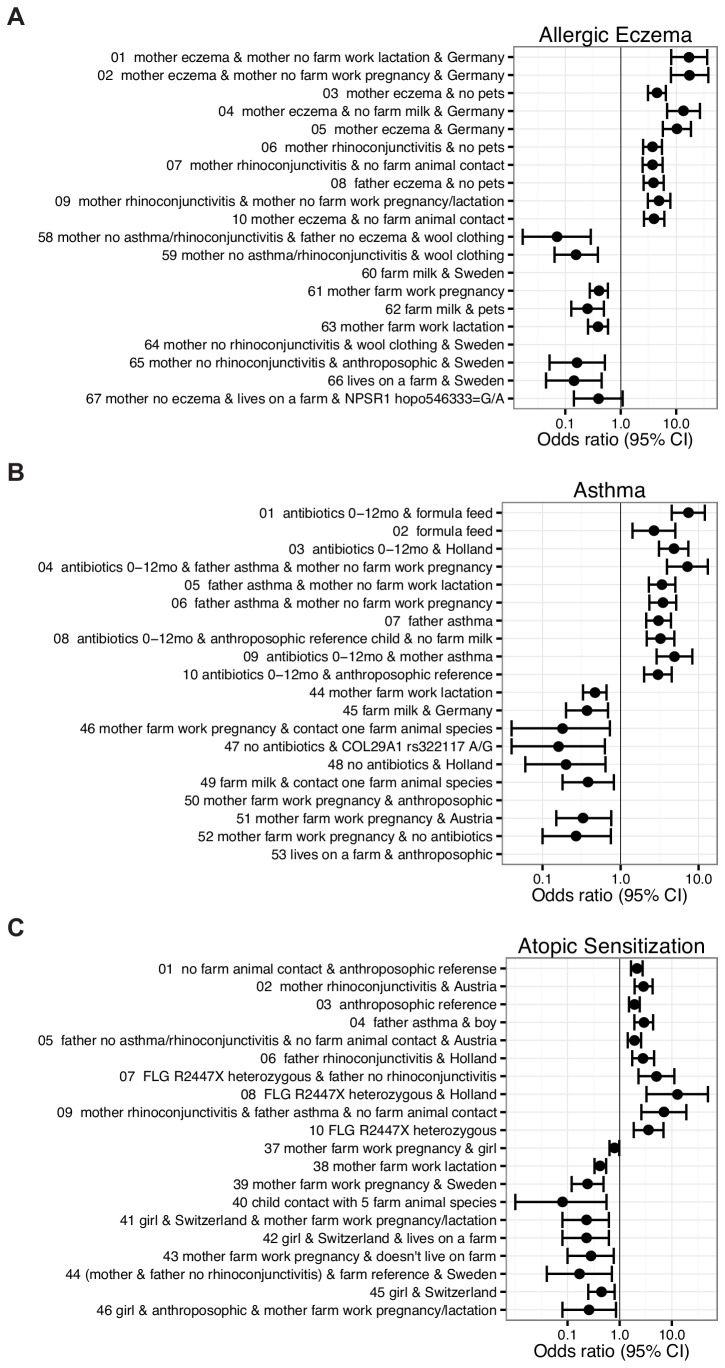
Combinations of genetic variants and/or environmental factors in relation to allergy and asthma in PARSIFAL. Odds ratios are shown for the top-hits rules identified for (**A**) allergic eczema; affected^1-10^ and unaffected^58-67^ (**B**) asthma; affected^1-10^ and unaffected^44-53^ and (**C**) atopic sensitization; affected^1-10^ and unaffected^37-46^. The odds ratios were calculated for children that fulfill all conditions in the rule using all other children as reference.

Many known risk and protective factors for allergic diseases were readily identifiable in the rule networks for allergic eczema including parental allergy in combinations with exposure to the farm environment or household pets early in life (Figure 3 A-B, Table S4-S5). For example, a markedly increased risk of developing allergic eczema was found if the mother had eczema and the child was not exposed to any farm animal (OR=4.0 CI 2.62-6.10, Figure 4 A and Figure 3 A; node V1 and J0). Alternatively, a strong protective effect with respect to allergic eczema was found if the mother had no history of asthma and/or rhinoconjunctivitis, the father had no history of eczema and the child wore wool clothing which reflects an anthroposophic lifestyle (OR=0.07, CI 0.02-0.29, Figure 3 B; node U2 and Z2). Furthermore, the number of different farm animal species could predict “affected” and “unaffected” children with respect to allergic eczema (Figure 3 A-B; node J0-J4). The SNPs did not appear as frequently as predictors in the networks; however, some top-ranked rules included genetic variants of *NPSR1* and *FLG* in combination with environmental factors. For example, a protective effect on *allergic eczema* was indicated among children living on a farm heterozygous (G/A) for hopo546333 in *NPSR1* with no history of maternal eczema (OR=0.39 CI 0.14-1.1).This genetic variant also appeared to prevent allergic eczema in conjunction with farm milk consumption during first year of life or if the mother worked on a farm during pregnancy and/or lactation (Table S5). Furthermore, we confirmed the well-established role of *FLG* mutations in eczema showing that German children in the PARSIFAL material with a 2282del4 deletion in the *FLG* gene had an increased risk to develop *allergic eczema* (OR=5.9, CI 2.7-12.9). This association was consistent in children from the other countries in the PARSIFAL study and the OR for all countries together was 2.3 (CI 1.2-4.2), which was also replicated in BAMSE (OR=2.6, CI 1.2-5.7).

#### Asthma

The top-ranked rules identified for childhood asthma includes both genetic and lifestyle factors, such as heredity, farm related lifestyle factors, the use of antibiotics in childhood, and if the child was formula fed or not (Figure 3 C-D, Table S6-S7). Individual rules in Figure 4 B, showed that the risk of developing asthma was significantly increased among children that had a father diagnosed with asthma, in combination with early use of antibiotics (<12 months), and if the mother was not exposed to the farm environment during pregnancy (OR=7.2, CI 3.9-13.1). On the other hand, children from the Netherlands that never used antibiotics displayed a protective effect (OR=0.20 CI 0.06-0.64). Other countries showed the same trend and the OR for all countries together was 0.24 (CI 0.14-0.42). A majority of the rules described for asthma showed that combinations of farm related lifestyle factors have a protective effect on childhood asthma. For example, if the mother worked on a farm during pregnancy or lactation period, and the child drank farm milk and was exposed to farm animals, than the child was protected from developing asthma (OR=0.18, CI 0.04-0.73). While children drinking farm milk were protected from asthma, we found an increased risk for developing asthma in children that were formula fed (OR=2.7, CI 1.4-5.0). Moreover, a combination of drinking baby formula and using antibiotics early in life (<12 months) suggested an even higher risk for developing asthma (OR=7.4, CI 4.5-12.0). We also found that children who never used antibiotics and were heterozygous (A/G) for a genetic variant in the *COL29A1* gene (rs322117) appeared to be protected from asthma (OR=0.15, CI 0.04-0.63).

#### Atopic sensitization

Among the factors found to affect the risk for atopic sensitization (IgE ≥3.5 kU/L) were parental history of allergy, farm environment exposure and if the child carried a *FLG* mutation (Figure 3 E-F, Table S8-S9). Also, heredity, country-of-origin and sex of the child appeared to be of importance. For example, in the top-ranked rules for atopic sensitization boys appeared in the “affected” group while girls were found in the “unaffected” group (Figure 4 C). Interestingly, we found that children carrying the *FLG* mutation R2447X had an increased risk of atopic sensitization (OR=3.6, CI 1.9-6.9), and the risk appeared even stronger if the child had a father with no history of rhinoconjunctivitis (OR=5.1, CI 2.3-11.2).

### Validation of Monte Carlo Feature Selection (MCFS)

Validation of the MCFS was performed once by repeating MCFS using only randomized data (220 factors) and once using both the original and randomized data (220+220 factors). On average 15.0 factors were found significant for each outcome in the original analysis (i.e. not including randomized data). When adding the randomized data, on average 14.2 of the original factors and 0.3 of the randomized factors were identified significant, suggesting that the addition of non-informative factors had a very small impact, if any, on the MCFS algorithm. When only the randomized factors were used for the MCFS, on average 1.5 factors were returned, verifying that few factors would be expected to be significant when dealing with non-informative data.

## Discussion

We have shown that interesting combinations of gene and environment factors with effects on allergy in children may be revealed by combining feature selection with machine learning. This approach has not earlier been used to study gene-environment interactions in relation to allergy in children. Factors identified as significant predictors of “affected” and “unaffected” in respect to allergic diseases were visualized in rule networks. Combinations of environmental and life style factors appeared most important for the development of childhood allergic diseases, while relatively few genetic variants reached the significance threshold. However, although the rule-based models containing genetic variants were weak on a global level, novel combinations of SNPs were suggested that influenced the risk of asthma in children.

Using the BAMSE and PARSIFAL studies, several predictive combinations were identified in one of the two datasets and then confirmed in the other. For example, we found an increased risk for *allergic asthma* related to a specific combination of genetic variants in *RORA* and *COL29A1*, and an increased risk for *current asthma* related to a specific combination of genetic variants in *ORMDL3*, *RORA* and *COL29A1*. Interestingly, both *RORA* and *ORMDL3* are among the asthma top-hit candidate genes identified by a large GWAS on asthma [6]. However, possible interactions between these genes in allergic diseases have not been previously described. The underlying mechanisms remain to be investigated, however it is intriguing to speculate that there may be a functional cross-talk between *ORMDL3* and *RORA* since both genes have been suggested a role in cellular stress response and lipid metabolism [33,34]. Furthermore, genetic variants in *NPSR1*, a well-studied asthma candidate gene, were found to increase the risk for *wheeze* in combination with *RORA*. This result supports recent work by Acevedo et al [35] which showed that *RORA* transcriptional levels may be regulated by the NPSR1 pathway. It should be noted that there were differences in allergy prevalence rates between the BAMSE and PARSIFAL studies, which is primarily explained by different background rates in the source populations. However, we believe that it is a strength of our work that the results were replicated in the two distinct materials. 

We also identified combinations of gene and environment factors that affected the risk of developing childhood allergic diseases, including variants of *COL29A1*. It has recently been discussed whether *COL29A1* is an eczema susceptibility gene [36] or not [37], but the interplay between this gene and lifestyle factors in allergic children have not been examined before. *COL29A1* is an epidermal collagen with the highest expression in skin, lung, and the gastrointestinal tract. Genetic variants in *COL29A1* may affect the epidermal barrier, resulting in an impaired skin or lung barrier, which is more vulnerable and sensitive to environmental allergens. Thus, the combination of *COL29A1* genetic variants and environmental exposures might be of high importance for the development of allergic disease and may explain the conflicting results regarding the role of *COL29A1* in eczema [36,37].

Moreover, the asthma candidate gene *NPSR1* appeared in conjunction with the environment to predict allergic eczema outcomes. Previous studies in PARSIFAL have shown that *NPSR1* haplotypes are moderately associated with asthma and atopic sensitization [38], but not eczema [39]. The results presented here agree with the findings by Bruce et al [13], also based on PARSIFAL, demonstrating that *NPSR1* can modify the effect of farming exposure on childhood allergy. 

Interestingly, several of the rules that predicted allergic outcomes based on a combination of genetic and environmental factors, involved heterozygous children. One explanation could be insufficient statistical power to detect a rare homozygous variant as a risk factor. Another and more intriguing hypothesis is that heterozygous children are more sensitive to certain environmental exposures. Children that are homozygous carriers of the risk allele, or the protective allele, may already be genetically programmed for an allergic or healthy outcome, respectively, and are thus less affected by combined exposure to certain environmental and lifestyle factors. Considering the example of *NPSR1*, we identified children heterozygous (A/G) for hopo546333 that combined with environmental factors were protected against allergic eczema. The homozygous group of children carrying the protective allele (GG) had enough power (n=2484) but were still not defined by this rule possibly because the effect of the protective allele overrides the environmental factors.

The most significant rules that came out of the analysis were those describing combinations of environmental and lifestyle factors. For example, early use of antibiotics increased the risk of asthma. Furthermore, we show that contact with farm animals, if the mother works on a farm during pregnancy or lactation and if the child consumes farm milk, all had a protective effect on allergic diseases. Earlier data on farm exposure and allergy suggested protective effects on asthma, rhinoconjunctivitis and atopic sensitization [40-42]. However, no clear connection was evident between farm exposure and eczema, although an inverse association between harvesting hay on a farm and eczema has been reported [43]. Importantly, we identified several factors related to farm environment exposure that had an effect on eczema susceptibility. Children living on a farm are exposed to a greater variety of environmental microorganisms compared to children not living on a farm, which may play an important role in modulating immune responses and thereby protecting against allergic diseases [44]. One hypothesis is that dust inhaled during farm work might act as carriers of protective agents, such as microbial antigens or immunomodulatory substances. In fact, Ege et al. [45] showed that harvesting hay is associated with higher gene expression of several *TLR* genes, which encode receptors of the innate immunity.

In this study we aimed at identifying patterns of interactions between genetic and/or environmental factors underlying allergic diseases. The materials under study have previously been analyzed using a limited number of factors, but no comprehensive analysis of the material has been performed to date. To achieve this, we used an innovative approach of feature selection and rule-based machine learning. Feature selection is usually necessary when dealing with a higher number of measurements (factors) than objects (children). It speeds up the learning process and increases the interpretability and generality of the models. The use of a multivariate feature selection method may aid the identification of feature dependencies [46]. In comparison to other feature selection methods, the MCFS focuses on finding factors useful for classification instead of, e.g., having the highest correlation to an outcome [30]. The use of machine learning methods, in particular the ability to examine a large number of factors simultaneously, have been suggested also by others to be valuable [26] and could help identify the underlying combinations that are important [47]. We used the ROSETTA system that produces rule-based models that are easy to interpret by non-experts. It describes combinations of values that are present in the original data, while many other machine learning methods (e.g. support-vector machines, neural networks) transform the data into functions making the model more difficult to interpret. Another difference compared to other modeling approaches (such as linear regression) is that although our rule-based models had low explanatory power we could still identify rules with high significance. The possibility to use these strong rules from a low-quality classifier demonstrated here is encouraging, since a high-quality classification might be impossible to achieve due to small effects of each factor and poorly defined phenotypes [23,48]. A limitation of exploratory data analysis is that the relations found do not necessarily imply causality and certain findings could be false positives. Our rules were tested for significance, and those p-values were Bonferroni corrected, which should greatly reduce the number of false positives. The use of two separate materials allowed for validation of individual rules and thus provided an independent estimate of rule quality. Furthermore, we have applied the MCFS algorithm to randomly generated data with similar statistical properties to assure that the selected factors are not a result of artifacts in the data. It should be stressed that the causality can only be assessed by hypothesizing about and testing the underlying biological mechanisms. Obviously, the conclusions from our study are limited by the genotype and environmental data available for analyses. GWA data were for example not included, and certain important candidate genes for asthma and allergy (e.g. *IL33* or *HLA-DQ*) were not assessed. Inclusion of other environmental exposures could also have affected the results. However, we believe we have included a sufficient number of important candidate genes and environmental exposures for allergic diseases in order to demonstrate the value of this analytical approach.

In conclusion, we describe a new bioinformatics approach to analyze and visualize complex data that may play a role in the development of allergic diseases. By combining feature selection and machine learning, important combinations of genes and/or lifestyle factors, related to the manifestation of allergic diseases, were identified. We believe that this approach is a useful tool when performing hypotheses-free analysis of large-scale datasets including genomic, epigenetic and other data. In fact, the methods that we used have a great potential to open a novel approach of analyzing genome-wide SNP data in the search for new gene-gene and gene-environment interactions underlying complex diseases such as asthma and allergy.

## Supporting Information

Methods S1 **Supplementary description of the methods.**
(DOC)

Table S1 **Overview of lifestyle- and environmental factors in PARSIFAL and BAMSE.** Name and description of all lifestyle- and environmental factors used in the analysis of gene-environment interactions in PARSIFAL by applying feature selection and rule-based classification. BAMSE data available for replication is indicated in the “BAMSE code column”.
(XLSX)

Table S2 **Overview of genetic factors in PARSIFAL and BAMSE.** An overview of 188 SNPs , representing 29 genes, genotyped in PARSIFAL. Overlapping SNPs (n=110) between PARSIFAL and BAMSE are indicated with “1” and used for gene-gene interaction analysis by MCFS and ROSETTA.
(XLS)

Table S3 **Rules based on the genetic factors.** An overview of all rules generated for the SNP data in PARSIFAL and BAMSE.
(XLSX)

Table S4 **Significant predictors (factors) selected by MCFS for *allergeniceczema*.** The displayed 24 factors were identified as significant (p < 2.3E-4) for the outcome *allergic* *eczema*.
(DOC)

Table S5 **Rules for the prediction of *allergiceczema*.** In total 83 rules were generated for *allergic* *eczema*, here presented with a unique identifier (‘Nr’).
(XLSX)

Table S6 **Significant predictors (factors) selected by MCFS for *asthma*.** The displayed 16 factors were identified as significant (p < 2.3E-4) for the outcome *asthma*.
(DOC)

Table S7 **Rules for the prediction of *asthma*.** In total 66 rules were generated for *asthma*, here presented with a unique identifier (‘Nr’).
(XLS)

Table S8 **Significant predictors (factors) selected by MCFS for *atopicsensitization*, >3.5 kU/liter.** The displayed 14 factors were identified as significant (p < 2.3E-4) for the outcome *atopic* *sensitization, >3.5* *kU/liter*.
(DOC)

Table S9 **Rules for the prediction of *atopicsensitization*.** In total 51 rules were generated for *atopic* *sensitization, >3.5* *kU/liter*, here presented with a unique identifier (‘Nr’).
(XLSX)
