# Supplementary material for: Rule-Based Models of the Interplay between Genetic and Environmental Factors in Childhood Allergy
Source: PLoS One. 2013 Nov 19;8(11):e80080. doi: 10.1371/journal.pone.0080080 (PMC3833974; doi:10.1371/journal.pone.0080080)
Supplement: Methods S1 — Supplementary description of the methods. (DOC) [file pone.0080080.s001.doc]

# Supplementary methods

*SNP Genotyping*

All genetic data available in our group (except GWA data), including 29 susceptibility genes for childhood allergies, was used in this study (Table S2). Primers for multiplex PCR and extension reactions were designed by the SpectroDesigner software (Sequenom GmbH, San Diego, CA, USA). PCR and extension reactions were performed according to manufacturer’s standard protocols. The SNP analysis was done by MALDI-TOF mass spectrometry (matrix-assisted laser desorption/ionization-time of flight; Sequenom GmbH). Each assay was validated using a set of 14 trios families, in total 42 individuals. Genotype data from these individuals are available through the HapMap consortium. Concordance analyses with the HapMap data as well as analysis of the parent-offspring-compatibility with the produced genotypes were performed. No significant deviation from Hardy-Weinberg equilibrium (P>0.05 using χ^2^ test) was seen for any of the SNPs and the average genotyping success rate was > 90%. For a few SNPs, genotyping was performed using TaqMan allelic discrimination assays according to the manufacturer’s recommendations (Applied Biosystems).

*Overview of the feature selection – machine learning methodology*

A feature selection algorithm, Monte Carlo feature selection (MCFS), was used to rank and select the SNPs and exposure factors that were significant as predictors for each phenotypic outcome. The factors identified as significant were then used for the machine learning part of the analysis.

The MCFS algorithm implemented in dmLab trains a large set of classifiers on small subsets (both of factors and children) of the original data set. For each of those classifiers, the factors are scored depending on their contribution to the classification. Finally, the factors are ranked according to the sum of these scores which is called their relative importance (*RI*). The details of the algorithm can be found in .

Machine learning methods are algorithms that automatically learn from data. Here, a rule-based classification method was used. In classification, the data set is typically divided into a training and a test set. The classifier is generated (trained) on the training set, and evaluated (tested) on the test set. The outcomes are known in the test set but not used in the prediction made by the classifier, and thus the model quality can be estimated by comparing the true outcomes with the predicted outcomes. We used classifier accuracy throughout the study, which is a measure of how likely the classifier is to predict the correct outcome for previously unseen data. The Rosetta software based on rough set theory was used to train a rule-based classifier.

Such a classifier is composed of IF-THEN rules. The IF part of the rule is a set of conditions, for example “no history of maternal eczema” and/or “heterozygote (G/A) for hopo546333”. The THEN-part shows the predicted outcome for objects (children) that match all conditions in the IF-part. For example one rule described a protective effect against allergic eczema:

“IF child lives on a farm AND heterozygote (G/A) for hopo546333 AND no history of maternal eczema THEN no allergic eczema”

For this rule there were 112 children in PARSIFAL that fulfilled all conditions in the IF-part. This number is called the rule support. All of those, 100 %, had the predicted outcome “no allergic eczema”. This fraction is called the rule accuracy. Support and accuracy was used to assess the quality of the rules, in addition to odds ratio and p-value.

The study was divided in two parts. Firstly, we aimed to find gene-gene interactions by constructing a data set with only SNPs (110 SNPs were available in both PARSIFAL and BAMSE) and the phenotypic outcome. For each material (PARSIFAL and BAMSE) feature selection and rule training was applied. The rules were analyzed, and as an independent validation the resulting rules were tested on the other material (PARSIFAL tested on BAMSE, and vice versa). Secondly, gene-environment interactions were tested by applying feature selection and rule training on all of the PARSIFAL material. PARSIFAL was chosen, as it contained the most exposure data. The analysis and rule validation was similar as to gene-gene, although not all rules could be tested on BAMSE, due to factors that were measured for PARSIFAL but not for BAMSE.

Detailed descriptions of the data processing, computational details and analysis are provided below.

*Dataset preparation*

Each disease variable, including asthma (current, ever, allergic- and non-allergic), eczema (allergic- and non-allergic), wheeze, hay fever and sensitization, was treated as one classification outcome (phenotype). Some of the genetic and exposure factors had missing values, and children with more than 20 % missing values were excluded from the analyses (when all PARSIFAL data was used between 63 and 87 children were removed, depending on the outcome, and when the SNPs common for PARSIFAL and BAMSE were used 1-15 children were removed from the PARSIFAL outcomes and 1 child from all outcomes in BAMSE).

The SNPs were transformed into a pseudo-binary representation ―with the possible values 0, 1 and 2―by counting the number of copies of the major allele. The *CCL1* SNP rs210839 had three alleles that were left untransformed and treated as nominal.

*Feature selection by MCFS*

DmLab 1.7 was used to run MCFS to rank the factors (factor overview in Table S1 and Table S2) and to calculate their significance for the prediction of the outcome (phenotype). We used a permutation test to assess the significance of the *RI* scores. The outcome values in the data table were randomly permuted 100 times to create new datasets which simulate a background distribution of the data, and the MCFS algorithm was run on each of these data sets. The calculation of *RI*s was repeated for each permuted data set, which gave 100 background scores for each factor. Using these scores the mean (*RI_f_^mean^*) and the standard deviation (*s_f_*) were calculated for each factor, *f*. The significance of a factor *f* was defined as the probability that the *RI* for *f*, *RI_f_*, came from a normal distribution with mean *RI_f_^mean^* and standard deviation *s_f_*. For the datasets with SNPs common between BAMSE and PARSIFAL (gene-gene) we also calculated an empirical p-value defined as the frequency of background *RI* scores that were lower than or equal to *RI_f_* for a given factor *f*.

The feature selection was done separately for each outcome and factors significant (p<0.05) after Bonferroni correction were kept. The Bonferroni correction was done by lowering the p-value threshold to 0.05/*n*, where *n* was the number of factors tested.

The settings for dmLab were: *projectionSize*=√*m*, *s*=10000, *t*=10, *u*=0, *v*=1, *balanceClasses*=TRUE, *balanceRatio*=1, and *cutPointRuns*=100. The parameters are explained in .

*Validation of feature selection using randomization*

The validation of the feature selection using MCFS followed a similar protocol as the MCFS. We used the original datasets―one for each outcome―prepared and organized as previously described. After removing children with more than 20 % missing values, however, we randomly re-sampled the factors values. The following procedure was done for each factor (column), *f_i_*, separately. First, a new factor (column) *R_f_i_* was created (‘R’ for ‘Random’ was added to the factor name). The frequency for each value for *f* was calculated, and for each child (data row) a value for *R_f_i_* was generated using probabilities derived from the observed frequencies. Two different types of validation experiments were performed: the first with the original factors, the randomized factors and the original phenotypes and another with only the randomized factors and a randomized phenotype.

*Training of the rule-based classifier*

Given a set of examples annotated with an outcome (here children with exposure and genetic data were annotated with an asthma and allergy phenotype) used for training, ROSETTA uses heuristics to find a minimal sets of factors―called reducts―that can discriminate equally well between all children as could all factors. These reducts are translated into IF-THEN rules which associate small sets of conditions (factors with specific values) with a particular classification outcome. Many such rules are trained, and together they can be used as a classifier, able to predict the outcome of objects that were not part of the training pipeline with previously unknown outcome. The classification is done by voting, in which each rule that matches an object casts a number of votes on an outcome based on the support of the rule in the training set. Finally, the classifier returns the outcome that has received the highest number of votes.

Undersampling was done prior to the rule training to prevent the uneven class distribution to affect the results. To prevent the undersampling to affect the model, the following strategy for undersampling was applied 100 times to create 100 similar datasets:

Let *n_i_* be the number of objects from outcome value *i*. Let *n_min_* be min(*n_1_*, *n_2_*, ..., *n_k_*), where *k* is the number of outcome values. Each object from outcome value *i* then have the probability *n_min_*/*n_i_* to be included in the dataset.

In our data we had two different outcome values (phenotypes): 1 = ”affected” and 2 = ”unaffected”. Following the strategy above, in average *n_min_* objects were selected from each outcome into each subset. Rules were trained on all subsets using the following algorithms implemented in ROSETTA. Firstly, the SNPs were discretized using the *EntropyScaler* algorithm. Missing values were replaced by their conditioned mean (the mean or for nominal factors the most common value among objects with the same phenotype) by the *ConditionedMeanCompleter* algorithm. Reducts were calculated using the *JohnsonReducer*, using approximate reducts and hitting fraction 0.9. The rules were generated from the reducts using the *RuleGenerator* algorithm. A filtering procedure was applied to remove rules with accuracy below 0.6 or support below 2.

Classifier accuracy was used to evaluate the classifier, and was defined as the number of correctly classified objects divided by all objects. A ten-fold cross validation scheme was used to test the classifier on other objects than those it was trained on. In the cross validation, the training set was treated with the same algorithms and settings as before. The test set was discretized using the discretization cuts found on the training set, and missing values were replaced by the mean or (if nominal values) the most common value using the *MeanCompleter* algorithm. The classification was done using the *StandardVoter* classifier, in which all applicable rules are allowed to vote for decision classes equal to their support, and the returned class is the one that received the highest number of votes. All ROSETTA algorithms used are described in the ROSETTA documentation.

The 100 rule sets that were created for each outcome were merged together. The accuracy and support of the individual rules were re-calculated using all available objects to reflect all the objects and to remove the undersampling.

*Rule terminology and quality measures*

The rule support was defined as the number of objects that match the left-hand side (LHS) of the rule, or equivalently the number of objects (*N*) multiplied with the probability that an object match the left-hand side (LHS) (Equation 1). The rule accuracy was defined as the fraction of the supporting children that also fulfilled the RHS, or equivalently the LHS-conditioned probability of *d*=*v* (Equation 2).

$Support= P(LHS)\cdot N$ (Eq. 1)

$Accuracy= P(d=v|LHS)$ (Eq. 2)

To evaluate the possible interaction effects in the rules we defined the expected accuracy (Equation 3) and expected support (Equation 4) derived as follows.

Let *U* be the set of all children. Then we define *T* as the set of all objects with the correct decision and *F* as the set of objects with the wrong decision:

$$T=\{x\in U:d(x)=v\}$$

$$F=\{x\in U:d(x)\neq v\}$$

Now, let *c_i_* be condition number *i* in the rule predecessor and *k_i_* be the value of *c_i_*. We then define *q_i_* as the objects that match *c_i_*=*k_i_* according to:

$$q_{i}=\{x\in U:c_{i}(x)=k_{i}\}$$

The probability that an element from *T* will match condition *i* is then:

$$p_{i}^{T}=p(x\in q_{i}|x\in T)=\frac{p(x\in q_{i}\cap x\in T)}{p(x\in T)}=\frac{\left|\{x\in q_{i}:x\in T\}\right|/\left|U\right|}{\left|T\right|/\left|U\right|}=\frac{\left|\{x\in q_{i}:x\in T\}\right|}{\left|T\right|}$$

Similarly, the probability that an element from *F* will match condition *i* is:

$$p_{i}^{F}=p(x\in q_{i}|x\in F)=\frac{\left|\{x\in q_{i}:x\in F\}\right|}{\left|F\right|}$$

Assuming that there are *n* conditions in the predecessor and that those are independent of each other, the conditions could be applied sequentially, and exactly$T\cdot p_{1}^{T}\cdot p_{2}^{T}\cdot\ldots\cdot p_{n-1}^{T}\cdot p_{n}^{T}$children from the correct outcome *v* and $F\cdot p_{1}^{F}\cdot p_{2}^{F}\cdot\ldots\cdot p_{n-1}^{F}\cdot p_{n}^{F}$from the other outcomes would match the rule. That would give the expected support and accuracy according to Equation 3 and Equation 4.

$ExpSupport= T\cdot p_{1}^{T}\cdot p_{2}^{T}\cdot\ldots\cdot p_{n-1}^{T}\cdot p_{n}^{T}+F\cdot p_{1}^{F}\cdot p_{2}^{F}\cdot\ldots\cdot p_{n-1}^{F}\cdot p_{n}^{F}$ (Eq. 3)

$ExpAccuracy = \frac{T\cdot p_{1}^{T}\cdot p_{2}^{T}\cdot\ldots\cdot p_{n-1}^{T}\cdot p_{n}^{T}}{ExpSupport}$ (Eq. 4)

Additionally, to evaluate the select the best rules we defined the following measures.

Ratio_PARSIFAL_ = (Support_PARSIFAL_*Accuracy_PARSIFAL_) / (ExpSupport_PARSIFAL_*ExpSuppport_PARSIFAL_) (Eq. 5)

Ratio_BAMSE_ = (Support_BAMSE_*Accuracy_BAMSE_) / (ExpSupport_BAMSE_*ExpSuppport_BAMSE_) (Eq. 6)

Ratio_Validation_ = “%1_match”_BAMSE_ / “%1_non-match”_BAMSE_ (Eq. 7)

where *Support* and *Accuracy* are defined according to Equation 1 and Equation 2, and *ExpSupport* and *ExpAccuracy* according to Equation 3 and Equation 4. The subscript indicates on what data the measures were done. The *“%1_match”* is defined as the percentage of outcome=1 (“affected”) among children that matched the rule and the *“%1_non-match”* is the similar percentage for those children that did not match the rule. Note that if the rule outcome is ”affected”, then the *“%1_match”* is equal to the accuracy.

*Cross-material validation of rules*

The datasets that were constructed to only contain SNPs present in both the PARSIFAL and the BAMSE material were used for rule validation. Rules trained on the BAMSE *gene-gene* data was tested on PARSIFAL and vice versa. In contrast, rules trained on the PARSIFAL data using both genetic and environmental factors (*gene-environment*) were tested on BAMSE only if they exclusively contained factors that were also available in BAMSE. The accuracy and support of the validated rules were calculated on the validation data according to Equation 1 and Equation 2.

The cross-material validation was done by comparing the percentage of children with the predicted phenotype among those that matched the LHS of the rule and those that did not match. If there was an effect in the predicted direction the rule was considered to be qualitatively correct. Children with missing data (needed for the rule) were excluded from the rule validation.

*Significance of rules*

The significance of the rules was calculated using the hypergeometric distribution as described by Equation 8.

$p(x;N,n,k)= \sum_{i=x}^{\min(k,n)} \frac{\left(\begin{matrix} k \\ x \end{matrix}\right)\left(\begin{matrix} N-k \\ n-x \end{matrix}\right)}{\left(\begin{matrix} N \\ n \end{matrix}\right)}$ (Eq. 8)

Where *x* is the number of children that match the LHS of the rule that had the predicted disease outcome, *N* is the number of children in the dataset, *n* is the number of children that match the LHS of the rule and *k* is the number of children in the dataset that have the predicted outcome. Hence, the p-value is the probability that at least the observed number of children that match the LHS of the rule would have the correct disease outcome by chance. We used the p-value threshold of 0.05. Bonferroni-correction, when applied, replaced the p-value threshold by 0.05/*n*, where *n* is the number of rules before rule filtering.

*Rule filtering*

After rule generation in ROSETTA we performed the following strategy for rule filtering:

Consider two rules, A and B, with the same outcome and the rule conditions *φ*_A_ and *φ*_B_ respectively. If *φ*_A_ is a subset of *φ*_B_, that is, if they contain the same conditions except for one or more that is present only in *φ*_B_, then rule B has been kept if and only if the p-value (hypergeometric distribution) of B is lower than that of A.

*Visualization of co-occurrences in rules*

We used a previously described strategy to visualize rule conditions (factors with values) that often co-occurred in rules. The pairs were scored by rule quality using the following strategy. Assume that *R*(*x,y*) is the set of rules that contained the conditions *x* and *y*. Then, the score is defined as (Equation 9)

$Score(x,y)=\sum_{r\in R(x,y)} Support(r)\cdot Accuracy(r)$ (Eq. 9)

The scores were calculated separately for the phenotypes ‘affected’ and ‘unaffected’. A rule network for each phenotype was produced using the Circos software for visualization of data and information . The conditions were positioned on the circle and two conditions were connected by a ribbon inside the circle if the pair had a non-zero score. The ribbons were formatted according to the scores using colors (the lower 75 % grey; the higher 25 % yellow to red-highest) and width (lowest score have less width).

For example, in Figure 3C, which summarizes rules for having asthma, there is a connection between "E 3" and "H 2" meaning "0-12 months when first used antibiotics" and "drank not mostly farm milk during first 12 months or no answer". When we checked, there was no rule that had only these two conditions, but four rules that had 1-2 other conditions in addition to these two. The additional conditions were A) from anthroposophic reference group, B) father had hay fever, C) mother never worked on farm during lactation and contact with two farm animal species, D) contact with two farm animal species.

Rule C) which is the most specific (four conditions) had the lowest support (31 children) and highest accuracy (32.3%). The other had accuracy between 22.1-22.2% and support 54-145.

The rule networks should be seen as a complement to the detailed analysis, as it gives an overall view of the rules in the classifier. Interaction terms can be seen as strong connections, but not all strong connections are interaction terms.

*Odds ratio calculations*

To estimate the disease risk for children classified by a specific rule, a new ‘exposure variable’ was created for each combination of genetic- and/or environmental factors identified by ROSETTA. For example, children were classified as *exposed* if homozygote for rs2305480 “GG” (major allele) and heterozygote for rs17270362 “GA”, and *non-exposed* (i.e. the reference group) if rs2305480 heterozygote, or homozygote for the minor allele and/or homozygote for rs2305480 minor or major allele. Logistic regression was used to test for associations between the “exposure variable” (i.e. the combination of factors identified by ROSETTA) and allergic outcomes using STATA. As the reference group, we used all children that were not carrier of all the specific genetic variants and/or exposed to the environmental factors described by the rule used for odds ratio calculation. The results are presented as ORs and 95% CI.

Furthermore, a dose-response analysis was performed for rule #6 and #9 (Figure 2C-D). Children were coded 1, if carrier of one risk-allele, 2 if carrier of two risk-alleles and 3 if carrier of three risk-alleles and then a logistic regression was used to test for a cumulative association between the number of risk alleles and *current asthma* or *wheeze*, respectively. The results are presented as ORs and 95% CI.

**References**

1. Draminski M, Rada-Iglesias A, Enroth S, Wadelius C, Koronacki J, et al. (2008) Monte Carlo feature selection for supervised classification. Bioinformatics 24: 110-117.

2. Komorowski J, Øhrn A, Skowron A (2002) The ROSETTA Rough Set Software System. In: Klösgen W ZJ, editor. Handbook of Data Mining and Knowledge: Oxford University Press.

3. Pawlak Z (1982) Rough sets. International Journal of Parallel Programming 11: 341-356.

4. Kooperberg C, Leblanc M, Dai JY, Rajapakse I (2009) Structures and Assumptions: Strategies to Harness Gene × Gene and Gene × Environment Interactions in GWAS. Statistical science : a review journal of the Institute of Mathematical Statistics 24: 472-488.

5. Hvidsten TR, Wilczyński B, Kryshtafovych A, Tiuryn J, Komorowski J, et al. (2005) Discovering regulatory binding-site modules using rule-based learning. Genome Research 15: 856-866.

6. Bornelöv S, Enroth S, Komorowski J (2012) Visualization of Rules in Rule-Based Classifiers. In: Watada J, Watanabe T, Phillips-Wren G, Howlett RJ, Jain LC, editors. Intelligent Decision Technologies: Springer Berlin Heidelberg. pp. 329-338.

7. Krzywinski M, Schein J, Birol İ, Connors J, Gascoyne R, et al. (2009) Circos: An information aesthetic for comparative genomics. Genome Research 19: 1639-1645.
