# Supplementary material for: Rule-Based Models of the Interplay between Genetic and Environmental Factors in Childhood Allergy
Source: PLoS One. 2013 Nov 19;8(11):e80080. doi: 10.1371/journal.pone.0080080 (PMC3833974; doi:10.1371/journal.pone.0080080)
Supplement: Table S4 — Significant predictors (factors) selected by MCFS for allergeniceczema. The displayed 24 factors were identified as significant (p < 2.3E-4) for the outcome allergic eczema. (DOC) [file pone.0080080.s005.doc]

**Table S4. Significant predictors (factors) selected by MCFS for *allergic eczema*.**

| **Rank** | **Factor** | **P-value** |
| --- | --- | --- |
| 1 | maternal eczema (yes/no) | <5E-324 |
| 2 | child drank mostly farm milk during first 12 months (yes/not yes) | 3.4E-222 |
| 3 | *FLG* R2447X | 1.4E-121 |
| 4 | mother worked on a farm during pregnancy (yes/no) | 1.1E-114 |
| 5 | country of origin (Sweden/Switzerland/The Netherlands/Germany/Austria) | 1.4E-94 |
| 6 | mother worked on a farm during pregnancy or lactation (yes/no) | 1.1E-92 |
| 7 | household pets during the first year of life (yes/no) | 3.8E-91 |
| 8 | paternal asthma and/or rhinoconjunctivitis (yes/no) | 6.9E-66 |
| 9 | mother worked on a farm during lactation (yes/no) | 3.4E-63 |
| 10 | paternal asthma (yes/no) | 9.1E-54 |
| 11 | group (lives on farm/from anthroposophic community/from farm reference group/from anthroposophic reference group) | 2.4E-53 |
| 12 | maternal asthma and/or rhinoconjunctivitis (yes/no) | 6.1E-53 |
| 13 | maternal rhinoconjunctivitis (yes/no) | 1.4E-48 |
| 14 | paternal eczema (yes/no) | 2.8E-47 |
| 15 | number of different farm animal species the mother had contact with during pregnancy (0-6) | 2.4E-36 |
| 16 | paternal rhinoconjunctivitis (yes/no) | 5.1E-25 |
| 17 | child lives on farm (yes/no) | 6.9E-21 |
| 18 | child wear/wore wool clothing directly on the skin (yes/no) | 1.2E-17 |
| 19 | maternal asthma (yes/no) (yes/no) | 1.5E-17 |
| 20 | number of different farm animal species the child had contact with (0-6) | 1.3E-13 |
| 21 | *FLG* S3247X | 5.4E-11 |
| 22 | *FLG* snp2282del4 | 1.2E-06 |
| 23 | *NPSR1* hopo546333 | 1.4E-05 |
| 24 | *TLR5* rs5744168 | 1.5E-04 |

The displayed 24 factors were identified as significant (p < 2.3E-4) for the outcome *allergic eczema.*
