# Supplementary material for: Rule-Based Models of the Interplay between Genetic and Environmental Factors in Childhood Allergy
Source: PLoS One. 2013 Nov 19;8(11):e80080. doi: 10.1371/journal.pone.0080080 (PMC3833974; doi:10.1371/journal.pone.0080080)
Supplement: Table S6 — Significant predictors (factors) selected by MCFS for asthma. The displayed 16 factors were identified as significant (p < 2.3E-4) for the outcome asthma. (DOC) [file pone.0080080.s007.doc]

**Table S6. Significant predictors (factors) selected by MCFS for *asthma*.**

| **Rank** | **Factor** | **P-value** |
| --- | --- | --- |
| 1 | use of antibiotics (never use/1st use>12 months/1st use 0-12 months) | <5E-324 |
| 2 | paternal asthma (yes/no) | 6.6E-122 |
| 3 | paternal asthma and/or rhinoconjunctivitis (yes/no) | 4.3E-65 |
| 4 | maternal asthma (yes/no) | 2.1E-51 |
| 5 | child drank mostly farm milk during first 12 months (yes/not yes) | 4.8E-37 |
| 6 | mother worked on a farm during pregnancy (yes/no) | 1.2E-33 |
| 7 | country of origin (Sweden/Switzerland/The Netherlands/Germany/Austria) | 2.3E-26 |
| 8 | paternal eczema (yes/no) | 3.7E-25 |
| 9 | group (lives on farm/from anthroposophic community/from farm reference group/from anthroposophic reference group) | 1.8E-14 |
| 10 | mother worked on a farm during lactation (yes/no) | 5.1E-13 |
| 11 | formula feed during babyhood (yes/no) | 6.4E-11 |
| 12 | paternal rhinoconjunctivitis (yes/no) | 1.7E-07 |
| 13 | child lives on farm (yes/no) | 1.5E-06 |
| 14 | maternal eczema (yes/no) | 4.4E-06 |
| 15 | *COL29A1* rs322117 (AA/AG/GG) | 2.4E-05 |
| 16 | number of different farm animal species the child had contact with (0-6) | 6.7E-05 |

The displayed 16 factors were identified as significant (p < 2.3E-4) for the outcome *asthma*.
