# Supplementary material for: Rule-Based Models of the Interplay between Genetic and Environmental Factors in Childhood Allergy
Source: PLoS One. 2013 Nov 19;8(11):e80080. doi: 10.1371/journal.pone.0080080 (PMC3833974; doi:10.1371/journal.pone.0080080)
Supplement: Table S8 — Significant predictors (factors) selected by MCFS for atopicsensitization, >3.5 kU/liter. The displayed 14 factors were identified as significant (p < 2.3E-4) for the outcome atopic sensitization, >3.5 kU/liter. (DOC) [file pone.0080080.s009.doc]

**Table S8. Significant predictors (factors) selected by MCFS for *atopic sensitization, >3.5 kU/liter*.**

| **Rank** | **Factor** | **P-value** |
| --- | --- | --- |
| 1 | number of different farm animal species the child had contact with (0-6) | 1.6E-228 |
| 2 | child lives on farm (yes/no) | 6.9E-95 |
| 3 | mother worked on a farm during pregnancy or lactation (yes/no) | 3.8E-91 |
| 4 | mother worked on a farm during pregnancy (yes/no) | 3.5E-73 |
| 5 | number of different farm animal species the mother had contact with during pregnancy (0-6) | 8.3E-71 |
| 6 | group (lives on farm/from anthroposophic community/from farm reference group/from anthroposophic reference group) | 3.6E-42 |
| 7 | mother worked on a farm during lactation (yes/no) | 3.4E-25 |
| 8 | *FLG* R2447X | 2.4E-18 |
| 9 | country of origin (Sweden/Switzerland/The Netherlands/Germany/Austria) | 6.4E-16 |
| 10 | paternal rhinoconjunctivitis (yes/no) | 1.9E-10 |
| 11 | maternal rhinoconjunctivitis (yes/no) | 2.2E-09 |
| 12 | sex (boy/girl) | 7.9E-09 |
| 13 | paternal asthma (yes/no) | 4.2E-05 |
| 14 | paternal asthma and/or rhinoconjunctivitis (yes/no) | 7.0E-05 |

The displayed 14 factors were identified as significant (p < 2.3E-4) for the outcome *atopic sensitization, >3.5 kU/liter*.
